# Genetic loci linked to Type 1 Diabetes and Multiple Sclerosis families in Sardinia

**DOI:** 10.1186/1471-2350-9-3

**Published:** 2008-01-20

**Authors:** Maristella Pitzalis, Patrizia Zavattari, Raffaele Murru, Elisabetta Deidda, Magdalena Zoledziewska, Daniela Murru, Loredana Moi, Costantino Motzo, Valeria Orrù, Gianna Costa, Elisabetta Solla, Elisabetta Fadda, Lucia Schirru, Maria Cristina Melis, Marina Lai, Cristina Mancosu, Stefania Tranquilli, Stefania Cuccu, Marcella Rolesu, Maria Antonietta Secci, Daniela Corongiu, Daniela Contu, Rosanna Lampis, Annalisa Nucaro, Gavino Pala, Adolfo Pacifico, Mario Maioli, Paola Frongia, Margherita Chessa, Rossella Ricciardi, Stanislao Lostia, Anna Maria Marinaro, Anna Franca Milia, Novella Landis, Maria Antonietta Zedda, Michael B Whalen, Federico Santoni, Maria Giovanna Marrosu, Marcella Devoto, Francesco Cucca

**Affiliations:** 1 Dipartimento di Scienze Biomediche, University of Sassari, 07100 Sassari, Italy; 2 Laboratorio di Immunogenetica, Ospedale Microcitemico, 09121 Cagliari, Italy; 3 Centro Sclerosi Multipla, Dipartimento di Scienze Neurologiche e Cardiovascolari, University of Cagliari, 09126 Cagliari, Italy; 4 Consiglio Nazionale delle Ricerche, Istituto di Neurogenetica e Neurofarmacologia, 09047 Cagliari, Italy; 5 Diabetologia Ospedaliera, Dipartimento di Medicina, Ospedale San Francesco, 08100 Nuoro, Italy; 6 Istituto di Clinica Medica, Servizio di Diabetologia, University of Sassari, 07100 Sassari, Italy; 7 Servizio di Diabetologia Pediatrica, Ospedale Brotzu, 09100 Cagliari, Italy; 8 Unità Operativa di Diabetologia, Ospedale Brotzu, 09100 Cagliari, Italy; 9 Dipartimento Pediatrico e Neonatologico, University of Sassari, 07100 Sassari, Italy; 10 Diabetologia Pediatrica Ospedale S. Francesco, 08100 Nuoro, Italy; 11 Diabetologia Pediatrica, Ospedale Crobu, 09016 Iglesias, Italy; 12 Prima Clinica Pediatrica, Unità Operativa di Diabetologia dell'Età Evolutiva, 09100 Cagliari, Italy; 13 CRS4, Parco Scientifico e Tecnologico, Polaris, Edificio 1, 09010 Pula, Italy; 14 The Children's Hospital of Philadelphia, Division of Human Genetics, and CCEB, University of Pennsylvania, Philadelphia, PA, USA; 15 Dipartimento di Medicina Sperimentale, University La Sapienza, 00185 Rome, Italy; 16 Sardegna Ricerche, Parco Scientifico e Tecnologico, Polaris, 09010 Pula, Italy

## Abstract

**Background:**

The Mediterranean island of Sardinia has a strikingly high incidence of the autoimmune disorders Type 1 Diabetes (T1D) and Multiple Sclerosis (MS). Furthermore, the two diseases tend to be co-inherited in the same individuals and in the same families. These observations suggest that some unknown autoimmunity variant with relevant effect size could be fairly common in this founder population and could be detected using linkage analysis.

**Methods:**

To search for T1D and MS loci as well as any that predispose to both diseases, we performed a whole genome linkage scan, sequentially genotyping 593 microsatellite marker loci in 954 individuals distributed in 175 Sardinian families. In total, 413 patients were studied; 285 with T1D, 116 with MS and 12 with both disorders. Model-free linkage analysis was performed on the genotyped samples using the Kong and Cox logarithm of odds (LOD) score statistic.

**Results:**

In T1D, aside from the HLA locus, we found four regions showing a lod-score ≥1; 1p31.1, 6q26, 10q21.2 and 22q11.22. In MS we found three regions showing a lod-score ≥1; 1q42.2, 18p11.21 and 20p12.3. In the combined T1D-MS scan for shared autoimmunity loci, four regions showed a LOD >1, including 6q26, 10q21.2, 20p12.3 and 22q11.22. When we typed more markers in these intervals we obtained suggestive evidence of linkage in the T1D scan at 10q21.2 (LOD = 2.1), in the MS scan at 1q42.2 (LOD = 2.5) and at 18p11.22 (LOD = 2.6). When all T1D and MS families were analysed jointly we obtained suggestive evidence in two regions: at 10q21.1 (LOD score = 2.3) and at 20p12.3 (LOD score = 2.5).

**Conclusion:**

This suggestive evidence of linkage with T1D, MS and both diseases indicates critical chromosome intervals to be followed up in downstream association studies.

## Background

T1D and MS are common inflammatory disorders which result from an autoimmune attack on the pancreatic beta-cells and the central nervous system, respectively. Both disorders are complex, multifactorial traits resulting from the interplay of largely unidentified predisposing genetic variants, in the presence of unknown environmental factors. Aside from the HLA region, only a few susceptibility variants have been detected in T1D, mainly using candidate gene, and more recently, genome wide association (GWA) strategies [1-4]. Until recently, no unequivocal identification of a non-HLA variant was reported in MS although recent work has provided consistent evidence that polymorphisms in the *IL7R *gene are associated with disease [5,6].

A number of whole genome linkage scans have been performed for both T1D and MS. These have mainly been based on the analysis of affected sib-pairs (ASPs), and have provided, overall, weak and conflicting results. The low power to detect small-size effect variants with low penetrance (typically exacerbated in linkage analysis) represents the most likely explanation for these failures. The likely presence of locus and pathogenic heterogeneity might have further complicated previous efforts. Contrast these disappointing results with the very recent successes of GWA studies [4,7] that are beginning to allow a more systematic understanding of some complex diseases. While the overall role of linkage analysis in multifactorial disease research appears to be modest, in principle some of the difficulties could be alleviated by further increasing the number of families in consortium-type studies [8]. However, this strategy, based on the analysis of thousands of families that have to be, perforce, collected from different populations, may actually result in an even higher degree of genetic heterogeneity and might still be underpowered to detect the kind of gene effects involved in multifactorial traits.

An alternative, still largely unexplored approach is to concentrate on large, genetically-isolated populations, such as Sardinia, where the diseases of interest are common and where there is evidence of powerful founder effects for all genetic systems so far studied. Notably, Sardinia represents the major exception to the general North-South gradient of both T1D and MS incidence in Europe. In Sardinia, T1D and MS not only have a much higher frequency compared with surrounding Mediterranean regions but they also show an increased probability of co-occurrence, in the same individuals and in the same families, which is only partially explained by shared genotype variation within the HLA complex [9]. The two disorders also show a correlated occurrence in other populations in which the main HLA associations are distinct and mutually exclusive [10]. This evidence suggests that susceptibility alleles at non-HLA immune-response loci might contribute significantly to the clustering of these two traits [9] and that they could be highly prevalent in Sardinia. Both the elevated frequency of these diseases and the fact that they are consistently prevalent throughout the island, that is, not restricted to certain subregions or communities, suggest that the genetic variants responsible are common and uniformly distributed and presumably not due to a large number of rare mutations. Furthermore, in this island population, reduced locus and pathogenic heterogeneity are expected; factors which have most likely impaired previous linkage efforts elsewhere. Considered globally, these factors suggest that, in Sardinia, some chromosome intervals containing variants for autoimmune diseases might be detected with linkage analysis, generating prior hypotheses to be further tested by means of association studies. In addition, positive evidence of linkage can also be used to weigh *P *values detected in GWA studies, thus helping to filter false positive results [11].

Here, we used a collection of Sardinian multiplex families with T1D and MS (including some in which both disorders were present in the same pedigrees) to perform a whole genome linkage scan.

## Methods

### Subjects

All families selected for this study had Sardinian origins dating back at least three generations from both the maternal and paternal side, as established by interviewing the parents when samples and informed consent were collected. The full dataset included 175 families with at least two affected individuals, which we refer to here as multiplex families, consisting of 413 affected individuals (285 with T1D, 116 with MS and 12 with both disorders) and a total of 954 samples (pedigrees of the collected families are provided in the online supplement). Since linkage analysis is informative only when there are at least two affected relatives in each pedigree, families with just one case affected by either T1D or MS (simplex) are effective only in the combined analysis of both diseases. As decomposition of the family dataset into the various subsets is complex, with some families containing both MS and T1D cases, a Venn diagram presenting the relationships is shown in Additional file 1. More specifically, for purposes of linkage analysis, the T1D sample set consisted of 120 multiplex families, including 105 two-generation nuclear families with at least two affected siblings (227 T1D patients) and 15 multicase, multigenerational families (62 T1D patients). Patients were selected to meet clinical criteria of T1D and to have disease onset before 35 years of age (average 13.1 ± 8.0 years, range from minimum 0.4 to maximum 34). This age of onset limit was used to maintain consistency with other linkage efforts, in particular with analyses performed by the T1D global consortium. The F/M ration in the T1D patients is 1.0. The MS sample set consisted of 58 families, including 54 two-generation families with at least two affected siblings (112 MS patients) and 4 multicase, multigenerational families (10 MS patients). The average age of onset of MS in the total sample set is 27.2 ± 9.1 years (ranging from minimum 9 to maximum 59) with a F/M ratio of 2.2. As shown in Additional file 1, there are 17 families in which cases of both pathologies are seen. These comprise 51 affected persons, 12 of whom have both diseases, 19 T1D, and 20 MS. In this combined diseases family dataset, the sex ratio is 21 F/10 M (2.1) for T1D, while it is 28 F/7 M (4.0) for MS and considering all patients, 38 F/13 M (2.9). In the families with only T1D, we see the expected slight male bias with an F/M ratio of 0.9 while in the MS families, the ratio is 1.7. These gender effects are consistent with those observed in much larger sample sets from the same population used in a previous analysis of HLA variants associated with these diseases [9]. Finally, in this combined diseases family dataset, the mean age of onset was 26.4 ± 8.4 years, range (13 – 40) for MS and 18.4 ± 10.7 years, range (7–33) for T1D patients.

Blood samples and clinical data for T1D patients were collected in the following Clinical Units: Divisione Pediatria, Ospedale G. Brotzu, Cagliari; Istituto di Clinica Medica, Servizio di Diabetologia, Università di Sassari; Diabetologia dell'Adulto, Ospedale Zonchello, Nuoro; Divisione Pediatria, Ospedale S. Francesco, Nuoro; Divisione Pediatria, Ospedale Crobu, Iglesias; Prima Clinica Pediatrica, Unità Operativa di Diabetologia dell'Età Evolutiva, Cagliari; Centro Diabetologico, Ospedale G. Brotzu, Cagliari.

Blood samples and clinical data for MS families were collected at the Multiple Sclerosis Centres in Cagliari. Forty-nine of these families had already been analysed in a previous linkage study [12]. The range and scope of the study were explained to all prospective participants. The study was approved by the Ethic Committee of the Universities of Cagliari and Sassari. All participating individuals and their parents or legal guardians signed a statement of informed consent.

### Genotyping

Genomic DNA was extracted from blood using standard salting-out procedures, after which it was coded and stored at -20°C until used.

A genome linkage scan was conducted for the whole sample set, initially using 474 microsatellite markers (398 purchased as commercial kits from Applied Biosystems: ABI PRISM LINKAGE MAPPING SETS V2.5 plus 76 additional markers, selected in the laboratory for those regions exhibiting prior evidence of linkage with T1D in other studies: chromosome 2q32.1-37.3, containing the *IDDM12 *and *IDDM13 *loci, the IDDM5 region of 6q16.1-q25.3, and 16q21-23.3 indicated in the large combined linkage study of Cox et al. [8,13-16]. After the first genome scan linkage analysis, 114 additional microsatellite markers were typed in the critical intervals showing evidence of linkage in the first phase of the study. More specifically we genotyped 31 additional markers from 1p31.1 only in the T1D families, 15 from 1q42.2 and 10 from 18p11.22 only in the MS families, as well as 36 from chromosome 10q21.1, 13 from 20p12.31 and 9 from chromosome 22q11.21 in both T1D and MS families.

Genotyping of microsatellites was carried out using a semi-automated method: fluorescently-tagged PCR products were separated on a polyacrylamide polymer using a MegaBACE 1000 DNA capillary sequencer. Microsatellites were amplified by PCR using fluorescently-labelled primers in a reaction volume of 7.5 μl (containing 5.25 pmoles of each primer, 1.5 nmoles of each of the four deoxynucleotide triphosphates and 0.2 units of Amplitaq Gold polymerase (Applied Biosystems) in a 2.5 mM MgCl_2 _buffer. PCR reactions were performed in MJ-PTC100 and MJ-Tetrad thermal cyclers using a standard file for all markers: 12' at 96°C, (30" at 94°C/30" at 55°C/30" at 72°C) × 10 times, (30" at 89°C/30" at 55°C/30" at 72°C) × 20 times, 10' at 72°C, 15°C ad infinitum. PCR was performed separately for each microsatellite marker which were then pooled in panels of 10–20 markers. After electrophoresis, the PCR fragments were sized and genotyped using the Genetic Profiler software (Amersham Biosciences). Alleles at each microsatellite were then given a numerical value (1, 2, 3, etc.) starting with the allele with the lowest number of base pairs.

### Statistical analysis

Before performing linkage analysis, genotype data were checked using the following procedures for quality control: 1) Mendelian inheritance was monitored using Pedstats [17] both for nuclear and extended pedigrees; 2) genetic distances between adjacent markers defined as the inter-marker recombination fraction and the relative order across the chromosome were computed from our data using the Best Order programme in the GAS package (GAS package version 2.3, (c) Alan Young, 1993–98) and cross-compared with available genetic maps (Généthon and Marshfield Clinic Center for Human Genetics) [18,19] and deCODE [20]. Any discrepancies were examined and, if deemed necessary, markers were retyped; 3) double recombinations within intervals of 30 cM were flagged and re-examined and when confirmed, were retained. Examination of double recombinations was performed using the MapMaker/SIBS programme [21]; 4) relative marker order was also compared with the available physical maps (University of California at Santa Cruz and Sanger Centre) [22,23]. Genotypes of markers showing inconsistencies between our genetic map and these physical maps were re-examined and, in two cases, the marker was discarded. After these quality control checks, only unequivocal genotype data were accepted while ambiguous data were removed. All linkage analyses were eventually performed using the genetic map [19], integrated when necessary with physical map information from the University of California at Santa Cruz [22].

To calculate the locus-specific sibling risk ratio for disease (as λs value), identity by descent sharing was estimated using the MapMaker/SIBS programme. For the purpose of this analysis, to estimate the proportion of alleles shared identical by descent in all available affected sib-pairs, complex pedigrees were decomposed into nuclear pedigrees using the Pedmanager programme [24]. To obtain confidence intervals for the λs values, we used the asymptotic method proposed by Cordell and Olson and Cordell and Carpenter [25,26].

Linkage of microsatellite markers with T1D and MS was evaluated in the individual disease sample sets and in the two datasets merged together (testing for linkage with an autoimmunity locus, treating each person with either disease as affected, and counting each family only once) using the Merlin software. Model-free multipoint linkage analysis was performed using the Kong and Cox modified version of the non-parametric linkage score to identify regions showing evidence for genetic linkage between markers and the presence of disease in families [27]. Allele frequencies were estimated by maximum likelihood using the Merlin software and including all individuals (the default option). Maximum LOD scores and corresponding nominal *P *values were calculated using the Kong and Cox linear model. Information content at various positions along the genome was estimated using Merlin. In order to perform genetic linkage analysis of markers on the X chromosome, we used the X-specific version of Merlin, Minx. Finally, exclusion mapping was performed at the canonical -2 LOD score value, for the hypothesis of λs = 2 and 3 by using the Genehunter software, version 2.1, release 3 [28].

## Results

**Figure 1:**
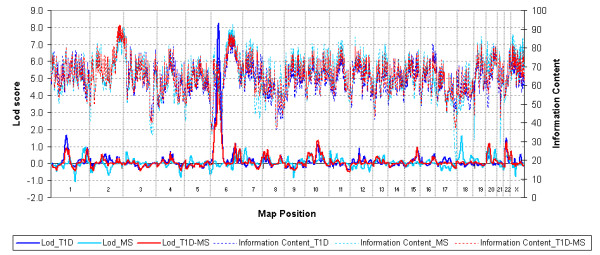
Non-parametric linkage analysis in T1D_SCAN, MS_SCAN and T1D-MS_SCAN. Reading from the left Y-axis, LOD score results are shown for each chromosome, proceeding from the short-arm telomere to the long arm telomere. The right Y-axis reports the corresponding information content for each point. The T1D and MS sample sets are described in the Materials and Methods section.

The mean distance between markers in the primary map of 474 microsatellites was 7.42 cM; the minimum distance was 0.01, while only three pairs of markers were separated by a gap of >20 cM. Results of the model-free linkage analysis with T1D, MS or both disorders on the whole genome, performed using the Merlin software [29] are shown in Table 1 and Figure 1. The T1D_SCAN (linkage analysis with Type 1 Diabetes) confirmed strong linkage to the HLA region (*IDDM1*), with a LOD score of 8.3 (*P *< 1.0 × 10^-5^). Estimation of the locus-specific disease risk for siblings of affected patients (λs) for *IDDM1 *was 2.81 (CI 1.56–5.07). Aside from the HLA region, we found four additional regions showing a LOD score ≥1: 1p31.1 (LOD 1.7, *P *= 3.0 × 10^-3^), 6q26 (LOD 1.1, *P *= 1.1 × 10^-2^), 10q21.2 (LOD 1.1, *P *= 1.3 × 10^-2^) and 22q11.22 (LOD 1.5, *P *= 4.0 × 10^-3^).

**Table 1:** Results of non-parametric linkage analysis on the whole genome implemented using Merlin software. Position (cM) refers to the genetic position of the maximum LOD score, while Position (bp) refers to the physical position given in build 36.1 of the genome (NCBI)

**Scan**	**Chromosome**	**Position(cM)**	**Position(bp)**	**Closest Marker**	**LOD**	**P Value**	**λs**	**CI**	**% Information_Content**
T1D_SCAN	1p31.1	109.48	82315937	D1S207	1.7	3.0 × 10^-3^	1.35	0.91 – 1.99	79
	6p21.32 (HLA)	40.48	32734820	D6S2447	8.3	< 1.0 × 10^-5^	2.81	1.56 – 5.07	71
	6q26	160.77	162759585	D6S1599	1.1	1.1 × 10^-2^	1.46	0.97 – 2.19	77
	10q21.2	78.64	64077501	D10S1652	1.1	1.3 × 10^-2^	1.33	0.89 – 2.00	70
	22q11.22	10.38	20587780	D22S539	1.5	4.0 × 10^-3^	1.89	1.05 – 2.57	66

MS_SCAN	1q42.2	246.27	232524978	D1S2800	1	2.0 × 10^-2^	2.23	1.06 – 4.70	61
	18p11.21	41.24	11482730	D18S53	1.6	3.0 × 10^-3^	3.2	1.28 – 7.99	64
	20p12.3	18.32	7607867	D20S115	1.1	1.3 × 10^-2^	1.8	0.93 – 3.47	76

T1D-MS_SCAN	6p21.32 (HLA)	39.65	32734723	D6S2447	5.1	< 1.0 × 10^-5^	1.65	1.15 – 2.34	69
	6q26	157.21	162115167	D6S305	1.2	9.0 × 10^-3^	1.25	0.93 – 1.69	79
	10q21.2	82.093	64077501	D10S1652	1.4	6.0 × 10^-3^	1.33	0.96 – 1.84	63
	20p12.3	18.32	7607867	D20S115	1.2	1.0 × 10^-2^	1.39	1.00 – 1.93	71
	22q11.22	10.38	20587780	D22S539	1.3	8.0 × 10^-3^	1.51	1.07 – 2.13	65

In the MS_SCAN (linkage analysis with Multiple Sclerosis), we found three regions showing a LOD score ≥1; 1q42.2, (LOD 1.0, *P *= 2.0 × 10^-2^), 18p11.21 (LOD 1.6, *P *= 3.0 × 10^-3^) and 20p12.3 (LOD 1.1, *P *= 1.3 × 10^-2^).

In the combined T1D-MS_SCAN (testing for shared autoimmunity loci) in which all families were analysed jointly, aside from the HLA, four regions showed a LOD >1 for shared autoimmunity loci, including 6q26 (LOD 1.2 *P *= 9.0 × 10^-3^), 10q21.2 (LOD 1.4, *P *= 6.0 × 10^-3^), 20p12.3 (LOD 1.2, *P *= 1.0 × 10^-2^) and 22q11.22 (LOD 1.3, *P *= 8.0 × 10^-3^). In this T1D-MS_SCAN, evidence of linkage in the HLA region was less significant (LOD 5.1, *P *= <1.0 × 10^-5^) than in the T1D_SCAN. This result reflects the modest genetic effect of the HLA region in the familiar clustering of multiple sclerosis.

Average information content for all scans was 66%.

Exclusion mapping for T1D and MS separately and, to find shared autoimmunity loci, jointly, was carried out testing all autosomes for the presence of a locus with a sibling relative risk of either 2 or 3. For T1D we could exclude 84% and 50% for a λs of 3 and 2, respectively. For MS, we could exclude only 41% and 29% for the presence of genes with a λs = 3 and 2. However, in the combined dataset of T1D and MS assessed jointly, 93% and 70% were excluded for the presence of loci with a λs = 3 or 2 (details are available on request).

Based on these results, we increased the density of markers in all intervals showing evidence of linkage ≥ 1, aside from chromosome 6q, due to the already high marker density in that region; results are shown in Table 2. This higher density of markers resulted in an increase of the information content, from an average of 68.3% to 84.1% in the critical intervals (see Table 1 and Table 2 for the information content of the individual regions in the successive mapping phases).

**Table 2:** Results on selected chromosome intervals after increment in marker density. Position (cM) refers to the genetic position of the maximum LOD score, while Position (bp) refers to the physical position given in build 36.1 of the genome (NCBI)

**Scan**	**Chromosome**	**Position(cM)**	**Position(bp)**	**Closest Marker**	**LOD**	**P Value**	**λs**	**CI**	**% Information_Content**
T1D_SCAN	1p31.1	109.43	82315937	D1S207	1.5	4.0 × 10^-3^	1.31	0.90 – 1.92	89
	10q21.1	77.943	61136296	D10S589	2.1	1.0 × 10^-3^	1.84	1.16 – 2.92	79
	22q11.21	10.38	20349111	D22S446	1.5	4.0 × 10^-3^	1.53	1.01 – 2.32	89

MS_SCAN	1q42.2	240.84	229780888	D1S251	2.5	4.0 × 10^-4^	4.03	1.44 – 11.3	93
	18p11.22	37.767	10732702	D18S1158	2.6	3.0 × 10^-4^	4.27	1.48 – 12.32	70
	20p12.3	15.43	6090696	D20S194	1.6	3.0 × 10^-3^	2.13	1.03 – 4.40	83

T1D-MS_SCAN	10q21.1	78.52	61136296	D10S589	2.3	6.0 × 10^-4^	1.58	1.13 – 2.24	83
	20p12.3	15.43	6090696	D20S194	2.5	4.0 × 10^-4^	1.77	1.22 – 2.56	81
	22q11.21	10.38	20349111	D22S446	0.9	2.0 × 10^-2^	1.26	0.93 – 1.70	90

In the T1D_SCAN, suggestive evidence of linkage was obtained after increasing the marker density in the 10q21.1 region with a maximum LOD score of 2.1 (*P *= 1.0 × 10^-3^) at marker *D10S589*. Estimation of the relative risk for 10q21.1 provided a λs of 1.84 (CI 1.16–2.92). For the 22q11.21 region, the maximum LOD score was unchanged, 1.5, but the position of the peak score moved to the nearby marker *D22S446 *(*P *= 4 × 10^-3^), while in the interval on chromosome 1p31.1 the maximum LOD score decreased to 1.5 at marker *D1S207 *(*P *= 4 × 10^-3^).

In the MS_SCAN, suggestive evidence of linkage was obtained using the enriched map at 1q42.2 at marker *D1S251 *(LOD = 2.5, *P *= 4.0 × 10^-4^) and at 18p11.22 at marker *D18S1158 *(LOD = 2.6, *P *= 3.0 × 10^-4^). The λs were 4.03 (CI 1.44–11.3) and 4.27 (CI 1.48–12.32) in these two intervals respectively. For the 20p12.3 region, we found a LOD score of 1.6 at marker *D20S194 *(*P *= 4.0 × 10^-3^) in these families.

Following analysis of the individual diseases, we then used the enriched marker maps in the intervals showing some initial evidence of linkage to a shared autoimmunity locus to conduct a joint T1D and MS scan. While there was a decrease in the LOD score (<1 at *D22S446*) in 22q11.21 when all T1D and MS families were analysed together, two regions showed suggestive evidence of linkage in this combined T1D-MS_SCAN. Notably, the 10q21.1 region showed a LOD score of 2.3 (*P *= 6.0 × 10^-4^) with a λs of 1.58 (CI 1.13 – 2.24) at marker *D10S589 *and the 20p12.3 region gave a LOD score of 2.5 (*P *= 4.0 × 10^-4^) with a λs of 1.77 (CI 1.22 – 2.56) at marker *D20S194*.

## Discussion and Conclusion

Using a collection of Sardinian multiplex families, we detected some suggestive evidence of linkage with T1D, MS and both disorders together, in specific chromosome intervals. In particular, the sample set of MS families gave suggestive evidence of linkage to chromosome 1q and 18p for which there were some prior claims of involvement in some other autoimmune or inflammatory pathologies in other populations [30-33].

Suggestive evidence of linkage with T1D was instead obtained on chromosome 10q21. This observation was somewhat reinforced by the joint analysis of families with either T1D or MS patients and thus might be indicative of a general autoimmunity locus. Consistent with this view, evidence of linkage to the same region of chromosome 10 was previously detected for Rheumatoid Arthritis [34,35]. Furthermore, linkage to 10q was also reported in a meta-analysis of MS whole genome linkage studies, although from the binning procedure used in the meta-analysis, it is difficult to determine with high-resolution if the peak is co-incident with ours [31].

**Figure 2:**
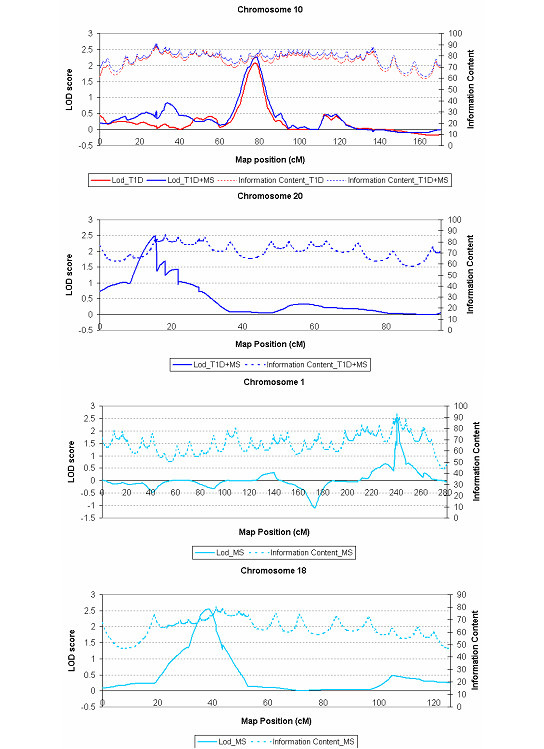
Non-parametric linkage analysis of chromosome intervals showing preliminary evidence of linkage after increasing marker density. The graphs refers to chromosome 10 in the T1D and T1D_MS scan, chromosome 20 in T1D_MS scan and chromosomes 1 and 18 in the MS scan. Reading from the left Y-axis, LOD score results are shown for each chromosome, proceeding from the short-arm telomere to the long arm telomere. The right Y-axis reports the corresponding information content for each point. The T1D, MS and combined T1D_MS sample sets are described in the Materials and Methods section.

Another locus to consider as potentially involved in both T1D and MS is the one we found on 20p12.3. What makes this interesting is that the locus was identified as being weakly significant for each disease considered individually, but only upon combining the analysis and increasing the information content in the region were we able to find the maximal signal and detect suggestive evidence of linkage. Linkage of this region to autoimmune disease has not been reported previously, but given the peculiar characteristics of the Sardinian population, this could be due to the presence of a founder autoimmunity variant, whose detection was in fact the underlying motivation of the study. The overall findings of the most promising regions for the two diseases following our typing of additional markers is shown in Figure 2. Several issues must be considered to give proper context to these findings.

One is that we did not see any evidence of linkage to the intervals containing variants detected in recent GWA scans in T1D [4], MS [5] and, in the case of the interleukin 2 receptor A on 10p15, in both diseases [5,36]. However, this is not unexpected, since these association signals, albeit statistically very significant, show genetic effects far below the threshold of detection by linkage analysis. It should be pointed out that our study was addressed to detect non-HLA genes with relevant effects in the familial clustering of the assessed diseases. For example, power calculation shows that with 234 affected sib-pairs, such as the size of the combined T1D_MS scan, we have >80% power to detect loci conferring a sibling risk ratio of >1.56 with a LOD score of 2 or more. These size effects could coincide with the areas of linkage detected in this study although, as in other linkage studies, our results do not allow unequivocal distinction between genuine evidence of linkage and random fluctuation in allelic sharing.

Furthermore, the key question remains: in the era of GWA scans, what is the role of linkage analysis to detect the genes contributing to the inherited risk of common multifactorial diseases? We believe that if the kind of genetic effects detectable with linkage analysis exist in these diseases, the founder population of Sardinia – where MS and T1D show among the highest incidences worldwide and tend to be co-inherited -could well be the venue to look for them using this cost effective strategy.

Hence, given the special characteristics of the Sardinian population and the opportunity to analyse families with both diseases jointly, the preliminary evidence of linkage to specific intervals is indeed promising, and can be refined in association analyses.

## Competing interests

The author(s) declare that they have no competing interests.

## Authors' contributions

MP performed microsatellite genotyping of T1D families and participated in microsatellite genotyping of MS families, performed statistical analysis on the data, revised the manuscript and designed all figures and tables. PZ helped in sample collection, supervised and participated in the microsatellite genotyping of T1D and MS families, collaborated in the statistical analysis and revised the manuscript. RM performed microsatellite genotyping of MS families, ED performed, with MP, the bulk of microsatellite genotyping of T1D families, MZ participated in microsatellite genotyping of T1D and MS families, DM collected blood samples and clinical data for T1D families, LM helped in sample collection, extracted DNA from peripheral blood and participated in microsatellite genotyping, CM helped with dataset construction and assisted in automating microsatellite genotyping. VO helped with the sample collection and DNA extraction, DC constructed the dataset of T1D families. GC, ED, ES, EF, LS, MCM, CM, ST, MR and DC participated in microsatellite genotyping of MS families. ML collected blood samples for MS families. MAS and SC extracted DNA from peripheral blood of MS families, RL participated in DNA extraction from peripheral blood, AN participated in DNA extraction. GP, AP, MM, PF, MC, RR, SL, AMM, AFM, NL and MAZ collected blood samples and clinical data for T1D patients. MW assisted in manuscript revision. FS helped with statistical analysis. MGM participated in the study design, led sample and clinical data collection from all MS patients and organised lab activities on the MS samples. MD participated in the study design, assisted in manuscript revision and supervised statistical analysis. FC designed and supervised the study and wrote the manuscript. All authors read and approved the final manuscript.

## Pre-publication history

The pre-publication history for this paper can be accessed here:



## Supplementary Material

Additional file 1 Family dataset. Set diagram of the families examined in this study. Because the two diseases may occur in the same family, and even in the same patient, there is overlap between the various family sets. The designation "Two generation" means that the families are nuclear families with at least two siblings affected by the respective disease. Multigenerational families are families in which there are at least two cases of the disease but the cases occur in different generations. Simplex families have only one case of a given disease (either MS or T1D) and are included here only when they present more than one affected child for the other disease or when considering both diseases together in the same family (searching for shared autoimmunity loci). For each designation, the minimal family structure with respect to each disease is considered. For example, 101 nuclear families with at least 2 siblings with T1D and no siblings with MS were studied, shown as Two generation T1D, as well as 1 nuclear family with at least 2 T1D and 2 MS patients (shown as the intersection of the Two generation T1D and Two generation MS sets, and 3 nuclear families with at least 2 T1D patients and one (simplex) MS case. Click here for file
