# Supplementary figures and images for: Genetic loci linked to Type 1 Diabetes and Multiple Sclerosis families in Sardinia
Source: BMC Med Genet. 2008 Jan 20;9:3. doi: 10.1186/1471-2350-9-3 (PMC2259316; doi:10.1186/1471-2350-9-3)

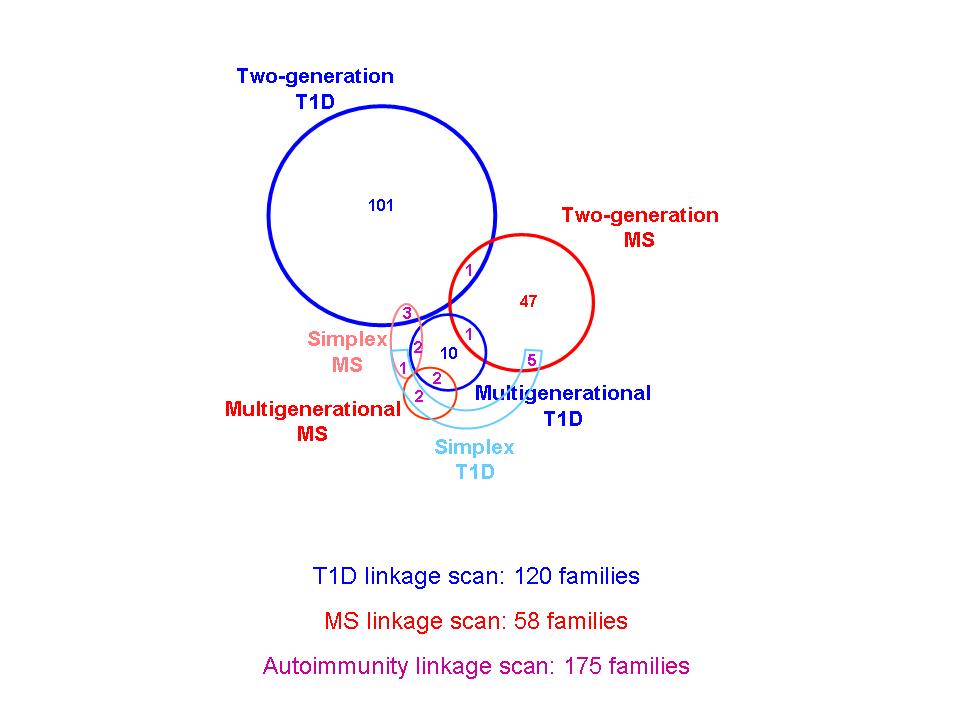

Supplement: Additional file 1 — Family dataset. Set diagram of the families examined in this study. Because the two diseases may occur in the same family, and even in the same patient, there is overlap between the various family sets. The designation "Two generation" means that the families are nuclear families with at least two siblings affected by the respective disease. Multigenerational families are families in which there are at least two cases of the disease but the cases occur in different generations. Simplex families have only one case of a given disease (either MS or T1D) and are included here only when they present more than one affected child for the other disease or when considering both diseases together in the same family (searching for shared autoimmunity loci). For each designation, the minimal family structure with respect to each disease is considered. For example, 101 nuclear families with at least 2 siblings with T1D and no siblings with MS were studied, shown as Two generation T1D, as well as 1 nuclear family with at least 2 T1D and 2 MS patients (shown as the intersection of the Two generation T1D and Two generation MS sets, and 3 nuclear families with at least 2 T1D patients and one (simplex) MS case. [file 1471-2350-9-3-S1.JPEG]
